# Excessive Neutrophil Extracellular Trap Formation Aggravates Acute Myocardial Infarction Injury in Apolipoprotein E Deficiency Mice via the ROS-Dependent Pathway

**DOI:** 10.1155/2019/1209307

**Published:** 2019-05-21

**Authors:** Zheliang Zhou, Shuning Zhang, Suling Ding, Mieradilijiang Abudupataer, Zhiwei Zhang, Xiaowei Zhu, Weiwei Zhang, Yunzeng Zou, Xiangdong Yang, Junbo Ge, Tao Hong

**Affiliations:** ^1^Shanghai Institute of Cardiovascular Diseases, Zhongshan Hospital, Fudan University, Shanghai 200032, China; ^2^Department of Cardiac Surgery, Zhongshan Hospital, Fudan University, Shanghai 200032, China; ^3^Department of Cardiology, Zhongshan Hospital, Fudan University, Shanghai 200032, China; ^4^Institutes of Biomedical Sciences, Fudan University, Shanghai 200032, China

## Abstract

Genetically human apolipoprotein E (*APOE*) *ε*32 is associated with a decreased risk of ischemic heart disease. *ApoE* deficiency in mice impairs infarct healing after myocardial infarction (MI). After the ischemic injury, a large number of neutrophils are firstly recruited into the infarct zone and then degrade dead material and promote reparative phase transformation. The role of ApoE in inflammation response in the early stage of MI remains largely unclear. In this study, we investigated the effect of *ApoE* deficiency on neutrophils' function and myocardial injury after myocardial infarction. By left coronary artery ligation in *ApoE* ^−/−^ and wild-type (WT) mice, we observed increased infarct size and neutrophil infiltration in *ApoE* ^−/−^ mice. Within the infarct zone, more neutrophil extracellular traps (NETs) were observed in *ApoE* ^−/−^ mice, while increased ex vivo NET formation was detected in *ApoE* ^−/−^ mouse-derived neutrophils through the NADPH oxidase-ROS-dependent pathway. Suppressing overproduced NETs reduced myocardial injury in *ApoE* ^−/−^ mice after ligation. In general, our findings reveal a critical role of apolipoprotein E in regulating Ly6G^+^ neutrophil activation and NET formation, resulting in limiting myocardial injury after myocardial infarction. In such a process, apolipoprotein E regulates NET formation via the ROS-MAPK-MSK1 pathway.

## 1. Introduction

Nowadays, the mortality due to cardiovascular disease still ranks as the top among all noncommunicable diseases globally, and the prevalence of ischemic heart disease keeps rising rapidly over the past two decades in China [1, 2]. The formation of atherosclerosis, the most common cause of ischemic heart disease, is due to low-density lipoprotein accumulation in the subendothelial space [3]. Thus, apolipoprotein E deficiency (*ApoE* ^−/−^) mice are wildly used in order to mimic and better understand this hypercholesterolemia-associated process.

Human *APOE* has three major allelic variants (*ε*2, *ε*3, and *ε*4), and most people carry at least one *ε*3. Genetically, the *ε*32 genotype is associated with a decreased risk of ischemic heart disease [4]. In addition to antioxidant activity found in neural cells (B12), ApoE was also reported to regulate hematopoietic stem cell proliferation through the promotion of cholesterol efflux [5, 6]. Current researches view *ApoE* ^−/−^ more as a tool to reach hypercholesterolemia conditions in vivo in order to better understand atherosclerosis or the myocardial ischemia process. The role of ApoE itself has not been fully investigated.

In human and experimental myocardial infarction (MI), inflammatory signals recruit a large number of neutrophils into the infarct zone within 24 h, and then, the number of neutrophils in the infarct zone reduces rapidly within 3 days. During this time, neutrophils along with other leukocytes degrade extracellular matrix constituents and help remove debris and macromolecules of injured cells [7]. Well-orchestrated inflammatory and anti-inflammatory progress can limit ischemic injury to the heart. However, such balance may be strongly disrupted and lead to adverse left ventricular remodeling [8]. The formation of neutrophil extracellular traps (NETs), a functional manner of neutrophils, acts as a defender of infection but a contributor to the pathogenesis of autoimmune-associated diseases as well [9]. Several kinds of research have shown a protective effect of targeting NET formation or extracellular DNA structure in the reperfusion model [10–12]. But there is little research reporting the role of NETs in a permanent MI model and the role of apolipoprotein E in such a process.

In this study, we aimed to investigate the role of apolipoprotein E in the pathogenesis of acute myocardial infarction (AMI) injury, especially its potential target, neutrophil, that takes part in ischemic injury. *ApoE* ^−/−^ mice were used to establish the AMI model for exploring the effect of *ApoE* deficiency on AMI injury and inflammation response, especially neutrophils and neutrophil extracellular traps within the infarct zone. Both animal and ex vivo experiment results showed that apolipoprotein E regulates NET formation through a ROS-dependent pathway. Notably, our results suggested that the number of neutrophils and NET formation regulates myocardial injury in the early stage of myocardial infarction, providing a promising target for minimizing infarction injury.

## 2. Materials and Methods

### 2.1. Animals and MI Model

Both male 6- to 8-week-old wild-type (WT) and *ApoE* ^−/−^ mice were bought from CAVENS Lab Animal. All animal protocols were approved by the Institutional Review and Ethics Board of Zhongshan Hospital of Fudan University.

Mice were anesthetized with isoflurane, bounded, trachea cannulated, and connected to a mechanical ventilator. The left side of the third or fourth intercostal was opened to expose the heart, and the left coronary artery was ligated to establish myocardial infarction (MI). Either apocynin (0.5 mg/kg, Selleck Chemicals) or Cl-amidine (25 mg/kg, Selleck Chemicals) was injected intraperitoneally 30 min before ligation in order to suppress NET formation. For control groups, the same volume of PBS was used.

### 2.2. Measurement of Infarct Size

Mice were euthanized with an overdose of sodium pentobarbital, and their hearts were then removed. Each heart was transversely sliced into 5 sections, stained in 0.5% 2,3,5-triphenyl tetrazolium chloride (TTC) under 37°C for 15 min. The noninfarct zone turned red, and the infarct zone turned white. Infarct size (%) was calculated as a percentage of the infarct area to total slice area.

Infarct size was further confirmed by the serum level of the CK-MB level (Nanjing Jiancheng Bioengineering Institute) or the cTnI level (Anogen).

### 2.3. Echocardiographic Analysis

Parasternal long-axis echocardiography was performed 7 days after ligation by Vevo® 2100. Briefly, mice were anesthetized with isoflurane and bounded onto a heating pad for maintaining body temperature. Parasternal long-axis M-mode echocardiography was recorded with the Vevo® 2100. Heart rate (HR), left ventricle ejection fraction (LVEF), and fraction shortening (FS) were calculated by the Vevo Lab Software.

### 2.4. Histopathological Analysis

Histopathology for neutrophil infiltration and NET formation was performed in the mice sacrificed 1 day after ligation. Hearts were excised and embedded in paraffin (Leica Biosystems) or in OCT (Sakura Finetek). Serial 5 *μ*m thick paraffin sections were used for Sirius red staining. Serial 5 *μ*m thick frozen sections were used for immunofluorescent costaining for NET formation (antihistone H3 citrulline R2+R8+R17 and Ly6G, Abcam). Costaining was visualized by fluorescence microscope with 488- and 596-conjugated secondary antibodies (BioLegend) mounted with Fluoroshield Mounting Medium with DAPI (Abcam). Neutrophils (magnification 200x) were quantified using five random Ly6G-positive fields per section and per animal. NET formation (magnification 400x or 200x) was quantified using five random cit-H3 (citrullinated histone H3) positive fields per section per animal. Percentages of the positive area were calculated in relation to the whole visual field with the ImageJ Software.

### 2.5. Single Cell Suspension and Flow Cytometry

Mice were sacrificed 1 day after myocardial infarction. Peripheral blood was drawn through the cardiac puncture into spray-coated K2EDTA tubes (BD Biosciences). Red blood cells were lysed with the RBC lysis buffer (BioLegend). And the remaining cells were resuspended with PBS containing 0.5% (wt/vol) BSA. The cells were incubated with the following antibodies: anti-CD11b-APC, M1/70 (BioLegend); -Ly6C-PerCP/Cy5.5, HK1.4 (BioLegend); and -Ly6G-PE, 1A8 (BioLegend). Neutrophils were identified as CD11b^+^ Ly6G^high^ Ly6C^low^. Data were acquired on the BD FACSAria™ II.

### 2.6. Quantitative Real-Time PCR

Total RNA was isolated, and the first-strand synthesis was carried out using the PrimeScript™ II 1^st^ Strand cDNA Synthesis Kit (Takara Bio Inc.). Quantitative real-time PCR using the TB Green™ Fast qPCR Mix (Takara Bio Inc.) was performed in a Bio-Rad CFX Connect™ Real-Time Detection System. Relative expression levels were determined by normalizing to Mus musculus *β*-actin (Actb) via the ΔΔC_t_ method.

The primer sequences used are given as follows: Actb, forward 5′-ACGTTGACATCCGTAAAGACC-3′, reverse 5′-ACACAGAGTACTTGCGCTCA-3′; Apoe, forward 5′-AACCGCTTCTGGGATTACCTG-3′, reverse 5′-GTCCTCCATCAGTGCCGTCA-3′; Tnf, forward 5′-CCCCTCTATTTATATTTGCACT-3′, reverse 5′-CCAAATCAGCGTTATTAAGACA-3′; Il6, forward 5′-CCTAGTGCGTTATGCCTA-3′, reverse 5′-GTCCCAACATTCATATTGTCA-3′; Mpo, forward 5′-GCCAAACTGAATCGCCAGA-3′, reverse 5′-ATGTTAAGAGCAGGCAAATCCA-3′; Elane, forward 5′-AGGCATTGACTCCTTCATCCG-3′, reverse 5′-CCTAGTTGGTCCTGCCCTCTCG-3′; and Itgam, forward 5′-CATTTCTTGCCTGTGACCAA-3′, reverse 5′-GTATTCTCAGCACCTAAACCC-3′.

### 2.7. Cell Isolation and NET Formation

Mice neutrophils were harvested and purified as previously described [13, 14]. In brief, bone marrow cells were gathered and added onto the Percoll gradient consisting of 52%, 65%, 78% Percoll layers and centrifuged at 2,500 rpm for 30 min at room temperature. The cell bands between 65% and 78% layers were harvested, and red blood cells were lysed with RBC lysis buffer. Cells were identified by flow cytometry with anti-CD11b-APC, M1/70 (BioLegend); -Ly6C-PerCP/Cy5.5, HK1.4 (BioLegend); and -Ly6G-PE, 1A8 (BioLegend).

Quantitation of NET formation was performed as previously described [15, 16]. Briefly, 0.5 × 10^5^ cells per well (6 to 8 duplicates per group) were plated into a black 96-well microplate (Corning). A NADPH oxidase inhibitor (apocynin, Selleck Chemicals), recombinant human APOE3 (PeproTech), P38 MAPK inhibitor (SB239063 or losmapimod, Beyotime Institute of Biotechnology), or ERK1/2 inhibitor (FR 180204, Beyotime Institute of Biotechnology) was pretreated 30 min before phorbol-12-myristate-13-acetate (PMA, Beyotime Institute of Biotechnology), Triton X-100 (Invitrogen), or H_2_O_2_ was added into wells according to protocol or demand. SYTOX Green (Invitrogen) was added 1 hour after PMA (100 nM) or H_2_O_2_ (8 *μ*M) treatment and incubated for 3 hours. Fluorescence was quantified at an excitation wavelength of 488 nm and an emission wavelength of 530 nm using an automated plate monochrome reader (FlexStation 3, Molecular Devices). NET formation was quantified by the fluorescence intensity analyses.

In order to quantify reactive oxygen species (ROS) generation, the same number of neutrophils was suspended in BSA-free and serum-free PBS. After 30 min with or without PMA treatment, DCFH-DA (Beyotime Institute of Biotechnology) was added and incubated for 30 min. Neutrophils were then resuspended with PBS and transferred into a black 96-well plate. Fluorescence intensity was quantified at an excitation wavelength of 488 nm and an emission wavelength of 530 nm using an automated plate monochrome reader. The number of cells was counted to normalize RFUs. In order to quantify ROS generation within infarcted hearts, newly harvested hearts were immediately embedded with OCT and sliced as soon as possible. Sections were incubated with PBS containing 5 *μ*M dihydroethidium (Beyotime Institute of Biotechnology) at 37°C for 30 min. And images were taken with a fluorescence microscope under the same exposure time. The mean gray value was quantified by the ImageJ software.

In order to virtualize NETs, 5 × 10^5^ cells per well were plated onto coverslips coated with poly-L-lysine. Fixed with 4% (wt/vol) paraformaldehyde 10 min after PMA treatment for 18 hours, DNA structures were stained with the primary anti-cit-H3 antibody and then with the 488-conjugated secondary antibody and DAPI. NETs were visualized by fluorescence microscope (Leica DM700). For life cell staining, 5 × 10^5^ cells per well were plated onto coverslips coated with poly-L-lysine. SYTOX Green and Hoechst 33258 (Beyotime Institute of Biotechnology) were added 3.5 h after PMA treatment. After being incubated for another 0.5 h, live cells were visualized by the fluorescence microscope.

### 2.8. Western Blotting

Neutrophils were harvested and divided into 3 subgroups (control, PMA, and PMA+APOE3). After being incubated with PBS, PMA, or PMA plus APOE3 for 1 hour, cells were centrifuged and lysed with RIPA buffer (Beyotime Institute of Biotechnology). Total protein was measured via the BCA method (Beyotime Institute of Biotechnology), and samples were boiled with InstantView™ SDS-PAGE loading buffer (Beyotime Institute of Biotechnology). Samples were separated by 12% SDS-PAGE gel (Beyotime Institute of Biotechnology) and transferred onto a PVDF membrane (Millipore). Total protein bands were visualized by using UV light (ChemiDoc™ Imaging System, Bio-Rad). Membranes were blocked with 5% (wt/vol) milk in TBST for 1 hour, then incubated with primary antibodies (Cell Signaling Technology) overnight. The blots were rinsed with TBST three times and incubated with HRP-conjugated secondary antibodies (Cell Signaling Technology) for 40 min. After being washed with TBST three times, the blots were applied to an ECL luminol reagent (Beyotime Institute of Biotechnology) and visualized via the ChemiDoc™ Imaging System. Bands were quantified by the ImageJ software.

### 2.9. Statistical Analyses

Results were expressed as the mean ± SEM of at least three independent experiments for each cell experiment group or at least six independent experiments for each animal group. Statistical tests included two-tailed Student's *t*-test, one-way analysis of variance followed by Tukey's multiple comparison test for parametric statistics, and Kruskal-Wallis test followed by Dunn's multiple comparisons for nonparametric statistics. A value of *p* < 0.05 was considered as statistically significant.

## 3. Results

### 3.1. Myocardial Injury Is Aggravated in *ApoE* ^−/−^ Mice after Ligation

**Figure 1:**
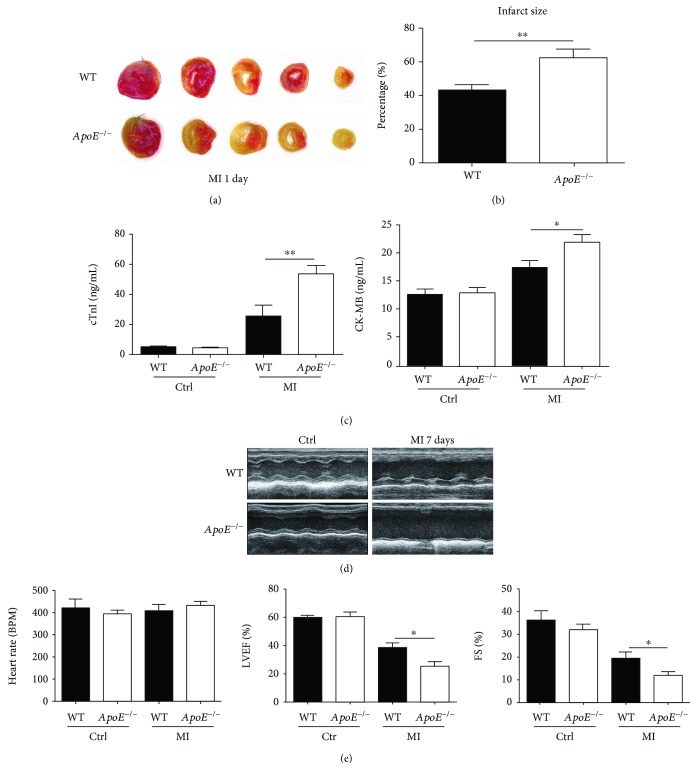
The role of *ApoE* deficiency in ischemic injury. (a, b) Representative TTC staining and quantitation of the infarct size of *ApoE* ^−/−^ and WT mice 1 day after ligation. Each group involves data of 6 mice. (c) Serum levels of cTnI and CK-MB of *ApoE* ^−/−^ and WT mice without surgery (Ctrl) or 1 day after ligation (MI). Each Ctrl group involves the data of 6 mice, and each MI group involves the data of 8 mice. (d, e) Representative echocardiographic images and quantitation of heart rate, left ventricle ejection fraction (LVEF), and fractional shortening (FS) of *ApoE* ^−/−^ and WT mice without surgery (Ctrl) or 7 days after ligation (MI). Each Ctrl group involves 6 mice, and each MI group involves 8 mice. Data are shown as mean ± SEM. Statistical tests include the two-tailed Student's *t*-test (two groups) and one-way analysis of variance followed by Tukey's multiple comparison test (more than two groups). ^∗^ *p* < 0.05 and ^∗∗^ *p* < 0.01.

To investigate the role of *ApoE* deficiency in myocardial infarction injury, we established myocardial infarction by permanent coronary artery ligation to both *ApoE* ^−/−^ and WT mice. The result of TTC staining showed that the infarct size was significantly larger in *ApoE* ^−/−^ mice compared to WT counterparts (Figures 1(a) and 1(b)). To further evaluate the myocardial injury, serum levels of cTnI and CK-MB were examined. In agreement with the TTC staining data, serum levels of cTnI and CK-MB increased significantly after myocardial infarction in both *ApoE* ^−/−^ and WT mice compared to control groups without surgery (Figure 1(c)). Additionally, serum levels of cTnI and CK-MB were significantly higher in *ApoE* ^−/−^ mice than in WT mice 1 day after ligation (Figure 1(c)). Furthermore, the echocardiography data indicated that the cardiac pumping function (LVEF and FS) was decreased in *ApoE* ^−/−^ mice than in WT mice 7 days after ligation, on the premise that the heart rate had no significant difference (Figures 1(d) and 1(e)).

Sirius red staining images showed a larger scar size of *ApoE* ^−/−^ mice compared to WT mice 7 days after ligation (Supplementary Figure 1a). Moreover, the survival curve showed that, at the late stage of healing (21 days or more), *ApoE* ^−/−^ mice had higher mortality after myocardial injury compared to WT mice (Supplementary Figure 1b). Our results indicated that *ApoE* deficiency aggravated acute ischemic injury after myocardial infarction, but such affection was not intense enough to increase the mortality during the acute inflammatory response stage.

### 3.2. *ApoE* Deficiency Exacerbates Neutrophil Activation after Myocardial Infarction

**Figure 2:**
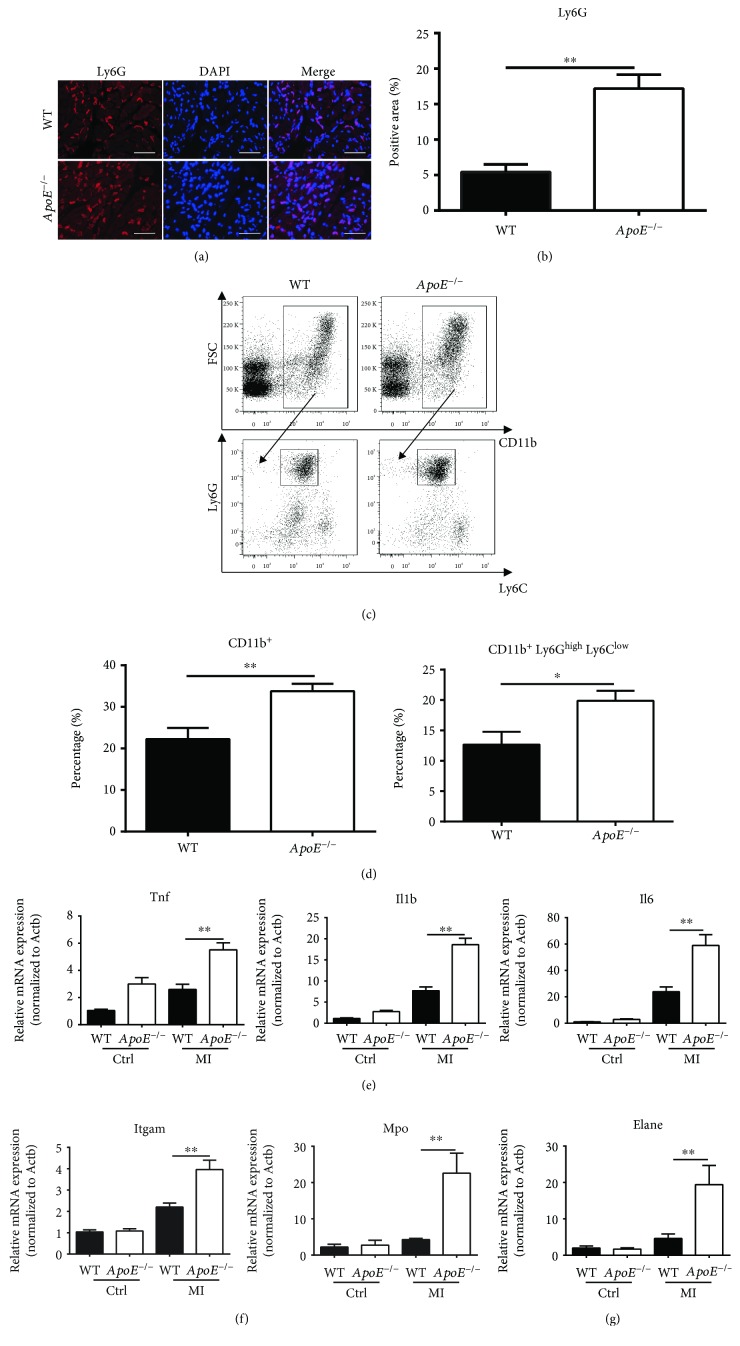
*ApoE* deficiency promotes neutrophil activation. (a, b) Representative immunofluorescent staining of Ly6G within infarcted hearts of WT and *ApoE* ^−/−^ mice 1 day after ligation and the percentage of the Ly6G-positive area of the whole image. Scale bars: 100 *μ*m. Each group involves 6 mice. (c, d) The gating strategy of flow cytometry and the percentage of myeloid cells (CD11b^+^) and neutrophils (CD11b^+^ Ly6G^high^ Ly6C^low^) in the blood of *ApoE* ^−/−^ and WT mice 1 day after infarction. Each group involves 8 mice. (e) Quantitative real-time PCR of proinflammation cytokines of tumor necrosis factor (Tnf), interleukin 1 beta (Il1b), and interleukin 6 (Il6) in infarcted hearts of *ApoE* ^−/−^ and WT mice without surgery (Ctrl) and 1 day after infarction (MI). Each group involves 6 mice. (f, g) Quantitative real-time PCR of integrin alpha-M (Itgam), neutrophil myeloperoxidase (Mpo), and neutrophil elastase (Elane) in infarcted hearts of *ApoE* ^−/−^ and WT mice without surgery (Ctrl) and 1 day after infarction (MI). Each group involves 6 mice. Data are shown as mean ± SEM. Statistical tests include the two-tailed Student's *t*-test (two groups) and one-way analysis of variance followed by Tukey's multiple comparison test (more than two groups). ^∗^ *p* < 0.05 and ^∗∗^ *p* < 0.01.

The inflammation response plays a double-edged role in ischemic injury and heart repair after infarction. Here, we hypothesized that *ApoE* deficiency might cause inflammation in infarcted hearts to develop in an unfavorable way. As we predicted, the result of immunofluorescent staining showed that the number of Ly6G-positive neutrophils increased significantly in the infarct and marginal zone of *ApoE* ^−/−^ mice compared to WT mice 1 day after ligation (Figures 2(a) and 2(b) and Supplementary Figure 2a). The result of flow cytometry (FCM) confirmed significant increases of CD11b^+^ myeloid cells and CD11b^+^ Ly6G^high^ Ly6C^low^ neutrophils in the peripheral blood of *ApoE* ^−/−^ mice compared to WT mice 1 day after ligation (Figure 2(c) and 2(d)). To further verify that *ApoE* deficiency enhanced the mobilization of immune cells after myocardial infarction, we detected the ratio changes in the blood of both mice before ligation and 3 days and 7 days after ligation by FCM. The data showed that the percentages of CD11b^+^ cells and CD11b^+^ Gr-1^+^ neutrophils (including CD11b^+^ Ly6C^+^ monocytes) were increased continuously in *ApoE* ^−/−^ mice than in WT mice after ligation (Supplementary Figure 2c and d).

To further examine the inflammatory response within infarcted hearts, we performed quantitative real-time PCR. The data showed that the mRNA expression levels of proinflammation cytokines—Tnf, Il1b, and Il6—were significantly upregulated in the infarcted hearts of *ApoE* ^−/−^ mice compared to WT counterparts 1 day after ligation (Figure 2(e)). Additionally, we detected the serum level of TNF-*α*. The ELISA result showed that the serum level of TNF-*α* was increased 1 day after ligation in both mice, while the level was significantly higher in *ApoE* ^−/−^ mice than in WT mice (Supplementary Figure 2b). The mRNA of integrin alpha-M (Itgam, CD11b) was upregulated in infarcted hearts of *ApoE* ^−/−^ mice compared to those of WT mice 1 day after ligation, indicating increased infiltration of CD11b^+^ cells within the infarcted hearts of *ApoE* ^−/−^ mice compared to those of WT mice (Figure 2(f)). Furthermore, quantitative real-time PCR results demonstrated a higher level of Mpo or Elane mRNA expression in the injured hearts of *ApoE* ^−/−^ mice compared to those of WT mice 1 day after infarction, indicating an increased function or number of neutrophils in the infarcted hearts of *ApoE* ^−/−^ mice (Figure 2(g)).

To confirm that apolipoprotein E deficiency caused the enhanced infiltration of neutrophils within infarcted hearts, we intravenously injected APOE3 into *ApoE* ^−/−^ mice 10 min before ligation. Immunofluorescent staining images of Ly6G showed that the percentage of the Ly6G-positive area was significantly decreased in the infarct zone of mice injected with APOE3 compared to that of PBS counterparts 1 day after ligation (Supplementary Figure 2e). The results demonstrated that apolipoprotein E deficiency played a causal role in the increased infiltration of neutrophils after myocardial infarction.

### 3.3. *ApoE* Deficiency Promotes NET Formation after Myocardial Infarction

**Figure 3:**
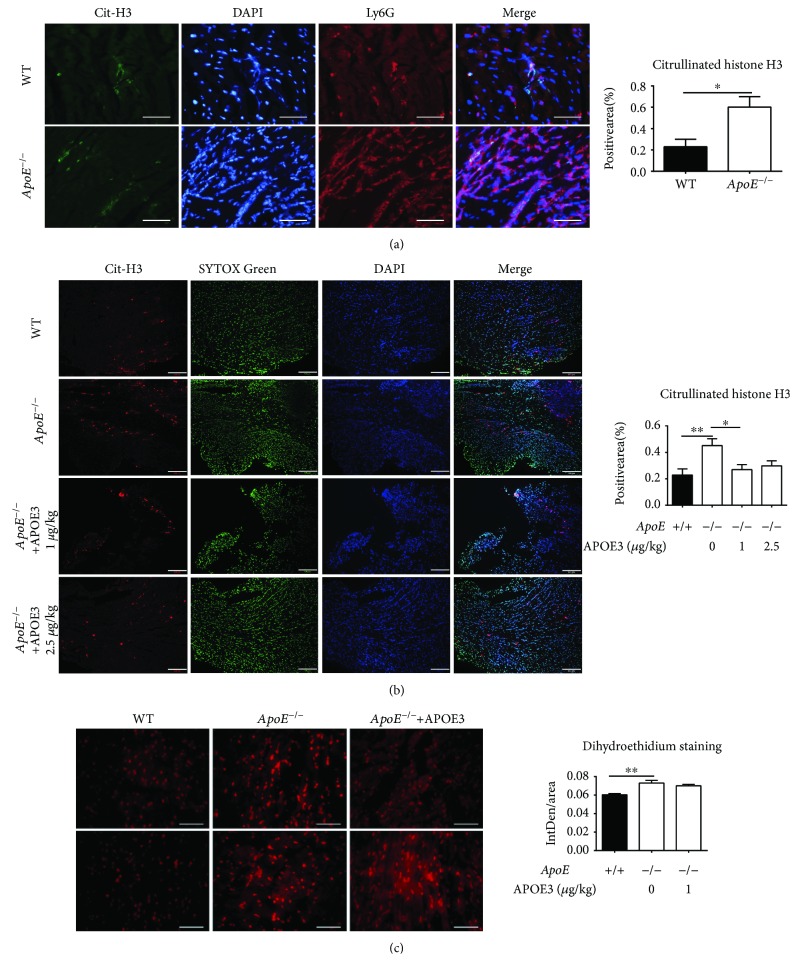
ApoE deficiency promotes neutrophil extracellular trap formation. (a) Representative immunofluorescent staining of anti-cit-H3 (anticitrullinated histone H3, green), Ly6G (red), and DAPI (blue) within the hearts of WT and *ApoE* ^−/−^ mice 1 day after ligation and the percentage of cit-H3-positive NETs of the whole image. Each group involves 6 mice. Scale bar: 50 *μ*m. (b) Representative immunofluorescent staining of anti-cit-H3 (red), SYTOX Green (green), and DAPI (blue) within the hearts of WT and *ApoE* ^−/−^ mice 1 day after ligation and the percentage of cit-H3-positive NETs of the whole image. Each group involves 6 mice. Scale bar: 200 *μ*m. (c) Representative dihydroethidium staining and quantitation of the mean gray value (integrated density/area) of the hearts of WT mice, *ApoE* ^−/−^ mice, and *ApoE* ^−/−^ mice injected with APOE3 1 day after ligation. Scale bar: 50 *μ*m. Each group involves 6 mice. Data are shown as mean ± SEM. Statistical tests include the two-tailed Student's *t*-test (two groups), one-way analysis of variance followed by Dunnett's multiple comparison test (b), and Kruskal-Wallis test followed by Dunn's multiple comparison test (c). ^∗^ *p* < 0.05 and ^∗∗^ *p* < 0.01.

To further explore the role of ApoE deficiency on neutrophil function, we then detected neutrophil extracellular traps (NETs), a functional manner of neutrophils, within infarcted hearts. Immunofluorescent staining images of citrullinated histone H3 (cit-H3, the marker of NETs) showed that the formation of NETs was significantly increased within infarcted hearts of *ApoE* ^−/−^ mice compared to those of WT mice 1 day after ligation (Figure 3(a)). Furthermore, most of the NETs were Ly6G positive (Figure 3(a)). In order to confirm the causal role of ApoE deficiency in NET formation, APOE3 was intravenously injected into *ApoE* ^−/−^ mice and immunofluorescent staining of cit-H3 was performed with hearts harvested 1 day after ligation. The result confirmed that APOE3 injection significantly reduced NET formation within the infarct zone of *ApoE* ^−/−^ mice (Figure 3(b)).

Given that the formation of cit-H3-positive NETs was ROS-dependent, ROS generation after the ischemic injury was then detected by dihydroethidium staining. The result showed that the ROS generation was increased within infarcted hearts of *ApoE* ^−/−^ mice compared to those of WT mice 1 day after ligation. Additionally, APOE3 treatment tended to decrease ROS generation within infarcted hearts of *ApoE* ^−/−^ mice though not reaching a statistical significance (Figure 3(c)).

### 3.4. *ApoE* Deficiency Promotes NET Formation through the NADPH Oxidase-ROS-Dependent Pathway

**Figure 4:**
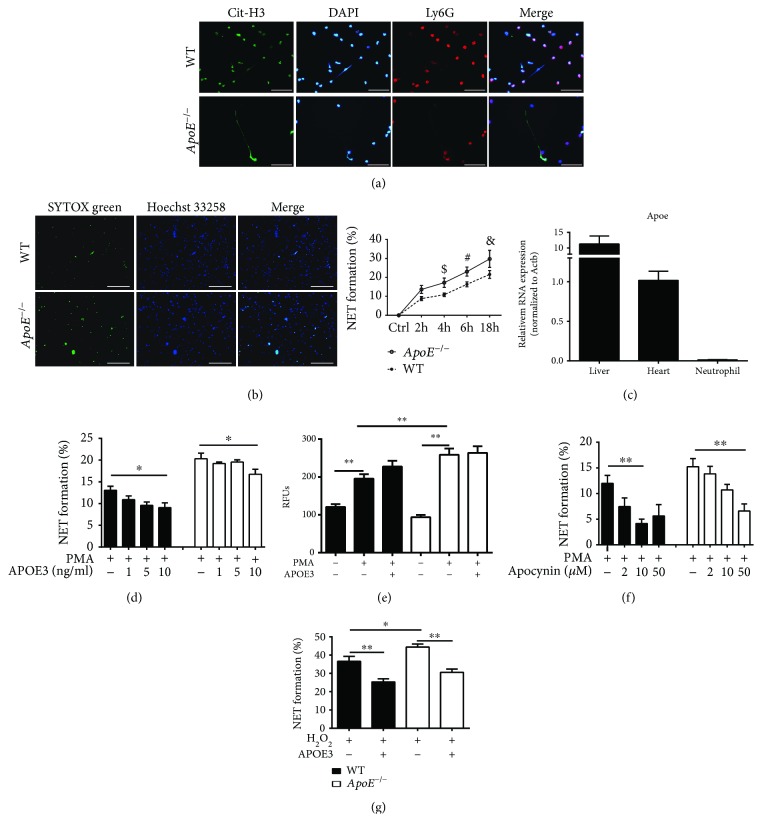
*ApoE* ^−/−^ deficiency regulates NET formation through the NADPH oxidase-ROS-dependent pathway. (a) Representative immunofluorescent staining of cit-H3 (green), Ly6G (red), and DAPI (blue) in *ApoE* ^−/−^- and WT-derived neutrophils treated with PMA for 18 hours. Scale bar: 50 *μ*m. (b) Representative live cell staining of SYTOX Green (green, extracellular DNA) and Hoechst 33258 (blue, total DNA) of *ApoE* ^−/−^- and WT-derived neutrophils stimulated with PMA for 4 hours. Scale bar: 100 *μ*m. And quantitation of ex vivo NET formation of *ApoE* ^−/−^- and WT-derived neutrophils stimulated with PMA for 2 h, 4 h, 6 h, and 18 h by SYTOX Green. ^$^ *p* < 0.05 (*ApoE* ^−/−^ mouse-derived neutrophils vs. WT mice 4 hours after stimulation), ^#^ *p* < 0.05 (*ApoE* ^−/−^ mouse-derived neutrophils vs. WT mice 6 hours after stimulation), and ^&^ *p* < 0.05 (*ApoE* ^−/−^ mouse-derived neutrophils vs. WT mice 18 hours after stimulation). (c) Quantitative real-time PCR of apolipoprotein E (Apoe) in the livers, hearts, and purified neutrophils of WT mice (*n* = 4). (d) Quantitation of ex vivo NET formation of *ApoE* ^−/−^ mouse- and WT mouse-derived neutrophils stimulated with PMA with or without APOE *ε*3 (APOE3) supplement for 4 hours. (e) Quantitation of ROS levels of *ApoE* ^−/−^ mouse- and WT mouse-derived neutrophils stimulated with PMA with or without APOE3 supplement for 4 hours. (f) Quantitation of ex vivo NET formation of *ApoE* ^−/−^ mouse- and WT mouse-derived neutrophils stimulated with PMA with or without apocynin supplement for 4 hours. (g) Quantitation of ex vivo NET formation of *ApoE* ^−/−^ mouse- and WT mouse-derived neutrophils stimulated with H_2_O_2_ with or without APOE3 supplement for 4 hours. Columns filled with black: WT mouse-derived neutrophils. Columns filled with white: *ApoE* ^−/−^ mouse-derived neutrophils. Data are shown as mean ± SEM. Statistical tests include the two-tailed Student's *t*-test (two groups) and one-way analysis of variance followed by Tukey's multiple comparison test (more than two groups) or by Dunnett's multiple comparison test (d, f). ^∗^ *p* < 0.05 and ^∗∗^ *p* < 0.01.

To figure out whether *ApoE* deficiency could directly promote NET formation, we performed an ex vivo experiment with neutrophils isolated from WT and *ApoE* ^−/−^ mice. The result of immunofluorescent staining showed that both kinds of neutrophils were cit-H3 positive after PMA treatment, in agreement with in vivo NET formation results (Figure 4(a)). More NET formation was detected in *ApoE* ^−/−^ mouse-derived neutrophils than in WT mouse-derived neutrophils after PMA stimulus (Figure 4(b)). The quantitative real-time PCR result showed that the expression level of Apoe was extremely low in neutrophil compared to the liver and heart (Figure 4(c)), suggesting that the primary cellular source of ApoE was from extracellular neutrophils. Furthermore, 10 ng/mL of APOE3 supplement could reduce NET formation in PMA-treated neutrophils isolated from WT or *ApoE* ^−/−^ mice (Figure 4(d)).

Given that cit-H3-positive or PMA-induced NETs were NADPH oxidase-ROS dependent, we first wondered whether APOE3 supplement could affect ROS generation. The result showed that PMA treatment promoted ROS generation in both neutrophils compared to untreated groups (Figure 4(e)). Although ROS generation was increased in *ApoE* ^−/−^ mouse-derived neutrophils after PMA treatment compared to WT mice, APOE3 failed to reduce ROS levels in both kinds of neutrophils after PMA treatment (Figure 4(e)). Pretreated with the NADPH oxidase inhibitor, apocynin, the results showed that PMA-induced NET formation was inhibited by apocynin treatment and confirmed that NET formation was NADPH oxidase-ROS dependent (Figure 4(f)). As APOE3 failed to suppress PMA-induced ROS generation, we then wondered whether APOE3 inhibited NET formation through the downstream signaling pathway of ROS generation. By using hydrogen peroxide (H_2_O_2_) treatment, we found that APOE3 could inhibit H_2_O_2_-induced NET formation in both kinds of neutrophils (Figure 4(g)). Additionally, NET formation was enhanced in *ApoE* ^−/−^ mouse-derived neutrophils after H_2_O_2_ treatment compared to WT mouse-derived neutrophils (Figure 4(g)). The results indicated that apolipoprotein E inhibited H_2_O_2_-induced or PMA-induced NET formation through downstream signals of the ROS pathway.

### 3.5. APOE3 Suppresses NET Formation via the ROS-MAPK-MSK1 Pathway

**Figure 5:**
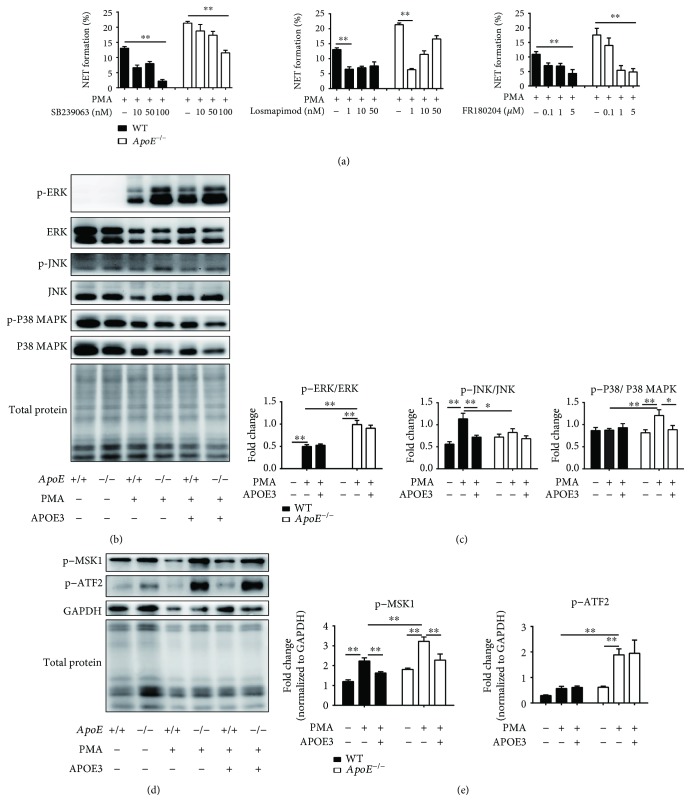
APOE3 affects NET formation through the MAPK-MSK1 pathway. (a) Quantitation of ex vivo NET formation of WT- and *ApoE* ^−/−^-derived neutrophils stimulated with PMA and different concentrations of the P38 MAPK inhibitor (SB239063 or losmapimod) or the ERK inhibitor (FR180204) for 4 hours. (b, c) Western blots and quantitation of the ERK1/2 (ERK), phosphorylated ERK1/2 (p-ERK), JNK, phosphorylated JNK (p-JNK), P38 MAPK, and phosphorylated P38 MAPK (p-P38 MAPK) expressions in WT- and *ApoE* ^−/−^-derived neutrophils stimulated with PBS, PMA, or PMA plus APOE3 for 1 hour. (d, e) Western blots and quantitation of the phosphorylated MSK1 (p-MSK1) and the phosphorylated ATF2 (p-ATF2) expression in WT- and *ApoE* ^−/−^-derived neutrophils stimulated with PBS, PMA, or PMA plus APOE3 for 1 hour. Columns filled with black: WT mouse-derived neutrophils. Columns filled with white: *ApoE* ^−/−^ mouse-derived neutrophils. Data are shown as mean ± SEM. Statistical tests include the one-way analysis of variance followed by Dunnett's multiple comparison test (a) and the Kruskal-Wallis test followed by Dunn's multiple comparison test (c, e). ^∗^ *p* < 0.05 and ^∗∗^ *p* < 0.01.

As the MAPK pathway was one of the downstream signals of the ROS pathway in NET formation, we investigated whether targeting MAPKs could suppress NET formation. The result of the NET formation experiment ex vivo revealed that both the P38 MAPK inhibitor (SB239063 or losmapimod) and the ERK1/2 inhibitor (FR180204) could significantly suppress NET formation in both *ApoE* ^−/−^ mouse- and WT mouse-derived neutrophils (Figure 5(a)). Western blotting data confirmed that after PMA treatment, the levels of phosphorylated ERK increased in both neutrophils, phosphorylated P38 MAPK increased significantly in *ApoE* ^−/−^ mouse-derived neutrophils, and phosphorylated JNK increased significantly in WT mouse-derived neutrophils (Figures 5(b) and 5(c)). Additionally, APOE3 treatment could inhibit PMA-induced ERK, JNK, and P38 MAPK phosphorylation in *ApoE* ^−/−^ mouse-derived neutrophils, but only phosphorylated P38 MAPK reached a significant decrease in *ApoE* ^−/−^ mouse-derived neutrophils while phosphorylated JNK reached a significant decrease in WT mouse-derived neutrophils (Figures 5(b) and 5(c)). The result indicated that ERK, JNK, and P38 MAPK are all involved in PMA-induced NET formation but only P38 MAPK was sensitive to apolipoprotein E deficiency.

To further figure out a possible target of APOE3, we then examined the levels of phosphorylated MSK1 and ATF2, downstream molecules of MAPKs, especially P38 MAPK. Western blotting data showed that PMA treatment increased MSK1 phosphorylation after PMA treatment in both kinds of neutrophils, while APOE3 treatment could decrease PMA-induced MSK1 phosphorylation in both kinds of neutrophils (Figures 5(d) and 5(e)). Moreover, the level of phosphorylated MSK1 was significantly increased in *ApoE* ^−/−^ mouse-derived neutrophils compared to each group of WT mouse-derived neutrophils with the same treatment (Figures 5(d) and 5(e)). The level of phosphorylated ATF2 increased after PMA treatment in both neutrophils compared to PBS treatment groups, but it only reached a significant increase in *ApoE* ^−/−^ mouse-derived neutrophils after PMA treatment. Additionally, APOE3 treatment had no significant effect on PMA-induced ATF2 phosphorylation in both mouse-derived neutrophils (Figures 5(d) and 5(e)). The increased levels of phosphorylated MSK1, phosphorylated ATF2, phosphorylated ERK, phosphorylated P38 MAPK, or phosphorylated JNK were in accordance with the increased NET formation (Figures 4(b) and 4(d)). These results suggested that MSK1 was a target of apolipoprotein E in regulating NET formation.

### 3.6. Suppressing NET Overproduction Reduces Myocardial Injury in *ApoE* ^−/−^ Mice

**Figure 6:**
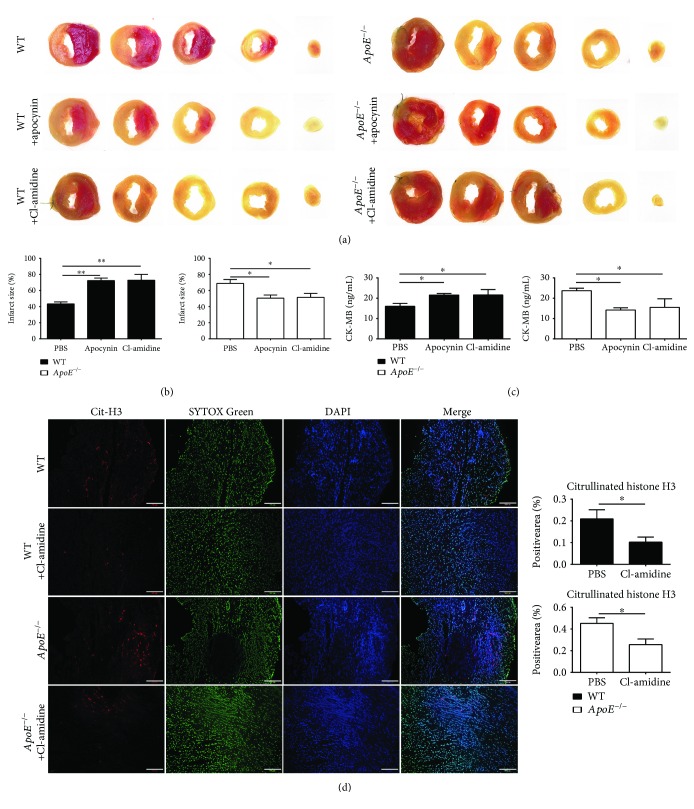
The role of excessive NET formation in myocardial infarction injury in *ApoE* ^−/−^ mice. (a, b) Representative TTC staining and the percentage of infarct size of WT or *ApoE* ^−/−^ mice with PBS, apocynin, or Cl-amidine injection 1 day after ligation. Each group involves 6 mice. (c) Serum CK-MB level of WT or *ApoE* ^−/−^ mice with PBS, apocynin, or Cl-amidine injection 1 day after ligation. Each group involves 8 mice. (d) Representative immunohistochemistry staining of cit-H3 (citrullinated histone H3, red), SYTOX Green (green), and DAPI (blue) within the hearts of WT or *ApoE* ^−/−^ mice with PBS, apocynin, or Cl-amidine injection 1 day after ligation. And the quantitation of the percentage of the cit-H3-positive area of the whole image. Scale bar: 100 *μ*m. Each group involves 6 mice. Columns filled with black: WT mice. Columns filled with white: *ApoE* ^−/−^ mice. Data are shown as mean ± SEM. Statistical tests include the two-tailed Student's *t*-test (two groups) and one-way analysis of variance followed by Dunnett's multiple comparison test (more than two groups). ^∗^ *p* < 0.05 and ^∗∗^ *p* < 0.01.

ApoE deficiency promotes NET overproduction through the NADPH oxidase-ROS-dependent pathway, and the formation of cit-H3-positive NETs is peptidyl arginine deiminase 4 (PAD4) dependent. Both the NADPH oxidase inhibitor, apocynin, and the PAD4 inhibitor, Cl-amidine, were used to clarify whether excessive NET formation was the direct cause of the increased myocardial injury in *ApoE* ^−/−^ mice. Apocynin (0.5 mg/kg) or Cl-amidine (25 mg/kg) was injected intraperitoneally 30 min before ligation to reduce NET formation in both *ApoE* ^−/−^ and WT mice. TTC staining data showed that the infarct size was decreased in the hearts of *ApoE* ^−/−^ mice injected with apocynin or Cl-amidine compared to those of *ApoE* ^−/−^ mice injected with PBS 1 day after ligation (Figures 6(a) and 6(b)). Interestingly, we observed that the infarct size was increased in WT mice after apocynin or Cl-amidine treatment (Figures 6(a) and 6(b)), suggesting myocardial infarction and repair need a proper number of NETs. Then, we evaluated myocardial injury by serum CK-MB concentration. Consistent with the TTC staining result, apocynin or Cl-amidine injection decreased the CK-MB level in the serum of *ApoE* ^−/−^ mice compared to that of *ApoE* ^−/−^ mice injected with PBS (Figure 6(c)). To verify the effect of Cl-amidine treatment, we performed immunofluorescent staining of cit-H3. Staining images of infarcted hearts showed that NET formation was significantly decreased after Cl-amidine treatment compared to PBS treatment in both *ApoE* ^−/−^ and WT mice (Figure 6(d)).

## 4. Discussion

In the present study, we demonstrate that excessive ROS generation and NET formation in infarcted hearts of *ApoE* ^−/−^ mice are accompanied with increased infiltration of CD11b^+^ Ly6G^high^ Ly6C^low^ neutrophils and activation of the NADPH oxidase-ROS-dependent pathway. *ApoE* deficiency promotes NET formation via the upregulation of ERK and P38 MAPK phosphorylation levels as well as the phosphorylation level of MSK1, a downstream molecule of MAPKs. However, APOE3 inhibits the formation of NETs not by directly inhibiting ROS generation but by regulating downstream of the ROS-dependent signal pathway, the MAPK-MSK1 signal pathway. Therefore, these data suggest a critical role for apolipoprotein E in alleviating myocardial injury by regulating neutrophil function and inflammatory response in the early stage of myocardial infarction.

Neutrophils take part in the first line of innate immunity progress triggered by invading pathogens or injury-induced pathogens [17]. Besides phagocytosis and oxidative burst, the formation of NETs is considered as an essential manner to defense infection [18]. After the ischemic injury, neutrophils are activated and recruited, participate in the inflammatory process, and remove debris of dead cells [19]. In addition to proinflammation functions, neutrophils also promote the differentiation and polarization of macrophages towards a reparative phenotype after myocardial infarction [20].

In addition to regulating circulating lipid homeostasis, apolipoprotein E possesses antioxidant activity in the neural cell line (B12) and regulates hematopoietic stem cell proliferation through promoting cholesterol efflux [5, 6]. Previous studies reported that *ApoE* deficiency promotes CD11b^+^ Ly6C^high^ monocytosis leading to impaired infarct repairment [21]. In our study, we also found increased ischemic injury in *ApoE*-deficient mice along with enhanced mobilization, infiltration, and extracellular trap formation of neutrophils. As NET formation has two enzyme-based mechanisms: peptidyl arginine deiminase 4 and neutrophil elastase [22], here, we found that the formation of NETs is through the ROS-dependent and citrullinated histone H3-dominant pathway after myocardial infarction. Interestingly, APOE3 inhibits NET formation but fails to significantly reduce ROS generation after acute treatment both in vivo and ex vivo.

PMA-induced NETs are NADPH oxidase-ROS dependent [5, 23] and can be abolished by the NADPH oxidase inhibitor, apocynin. Here, we found that a low dose of APOE3 suppressed both PMA-induced and H_2_O_2_-induced NET formation in *ApoE^−/−^* mouse- and WT mouse-derived neutrophils, which agrees with the previous study about the antioxidant activity of ApoE [5, 23]. However, the current study found that APOE3 treatment fails to reduce ROS generation after PMA treatment. Therefore, we speculated that APOE3 suppresses NET formation through the downstream signal of the ROS pathway. Previous studies reported that the Raf-MEK-ERK pathway and the P38 MAPK pathway are downstream of the NADPH oxidase-ROS-dependent pathway for NET formation [15, 24, 25]. Our data suggested that either the P38 MAPK or the ERK1/2 inhibitor treatment could suppress PMA-induced NET formation in both *ApoE^−/−^* mouse- and WT mouse-derived neutrophils. Furthermore, MAPK phosphorylation levels increased after PMA treatment in both kinds of neutrophils, but the APOE3 treatment only significantly decreased PMA-induced P38 MAPK phosphorylation in *ApoE^−/−^* mouse-derived neutrophils. These results demonstrated that MAPKs are involved in PMA-induced NET formation, but only P38 MAPK is sensitive to apolipoprotein E deficiency after PMA treatment. Thus, downstream molecules of MAPKs, especially P38 MAPK, have been detected. We found that the phosphorylated MSK1 expression increased significantly after PMA treatment while APOE3 treatment could decrease PMA-induced MSK1 phosphorylation in both kinds of neutrophils. Moreover, the level of phosphorylated MSK1 was significantly higher in *ApoE* ^−/−^ mouse-derived neutrophils compared to each WT mice counterpart, in accordance with the number of NET formation ex vivo. As inhibitors of MAPKs can inhibit MSK1 phosphorylation, we speculated that apolipoprotein E inhibits NET overproduction by reducing MSK1 phosphorylation [26]. But the underlying mechanism for APOE3 regulating MSK1 phosphorylation needs to be unraveled. And more selective MSK1 inhibitors are needed to further confirm whether targeting phosphorylated MSK1 could truly suppress NET formation.

Several studies have found that targeting NETs limits ischemia-reperfusion injury in WT mice or rats by reducing thrombosis or nonreflow [10, 11, 27]. For ST-segment elevation acute myocardial infarction (STEMI) patients, they may have an incidence of 5 to 50% suffering nonreflow after primary percutaneous coronary intervention [28], indicating that permanent ischemic injury weighs much in myocardial infarction. But the role of NETs in permanent myocardial infarction injury and repair after permanent ligation was not revealed. Here, we found that either apocynin or Cl-amidine treatment limits myocardial injury in *ApoE^−/−^* mice after acute myocardial infarction, indicating that excessive NET formation was one of the causes of increased myocardial injury. But as for WT mice, either apocynin or Cl-amidine treatment increased ischemic injury after ligation, which was entirely different from the ischemia-reperfusion model. The pathological process of the permanent ligation model may be affected less by thrombosis or hemodynamic changes beneath the ligation suture and may be affected mainly by hypoxia and inflammatory response, while thrombosis and hemodynamic changes could directly affect infarct size in the ischemia-reperfusion model [29]. Therefore, we speculate that a proper number of NETs is demanded after myocardial infarction. As most of the clinical patients suffering from AMI are accompanied with risk factors of promoting NET formation [30, 31], we consider that there will be a potentially beneficial effect of inhibiting NET formation for STEMI patients by both reducing nonreflow and limiting overactivated inflammatory response.

## 5. Conclusions

**Figure 7:**
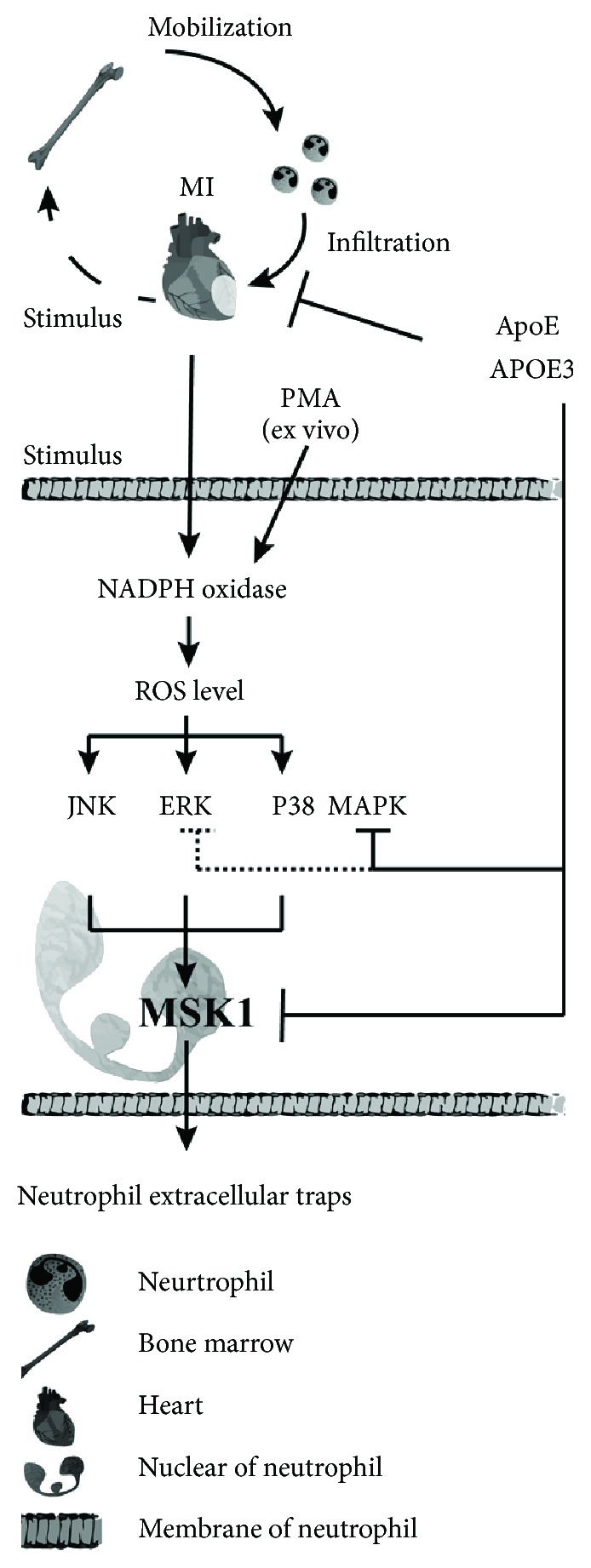
Schematic model of the role of ApoE/APOE3 in regulating neutrophil function, especially NET formation, in the context of acute myocardial infarction. After acute myocardial infarction, neutrophils are mainly mobilized from the bone marrow, infiltrate into the infarcted heart, and form NETs via the NADPH oxidase-ROS-MAPK-MSK1 signal pathway. In this process, apolipoprotein E regulates neutrophil infiltration and NET formation. As for NET formation, apolipoprotein E fails to reduce ROS generation but can inhibit MSK1 phosphorylation. Additionally, P38 MAPK is sensitive to apolipoprotein E deficiency. The APOE3 supplement inhibits P38 MAPK phosphorylation under the conditions of apolipoprotein E deficiency. Moreover, ApoE deficiency promotes PMA-induced ERK1/2 phosphorylation, but APOE3 fails to inhibit ERK1/2 phosphorylation after PMA treatment.

Our study demonstrates that lack of apolipoprotein E promotes NET formation via the ROS-MAPK-MSK1 pathway, leading to increased myocardial injury after myocardial infarction (research diagram in Figure 7). NET formation is activated by increased ROS generation in the early stage of myocardial infarction. The suppression of NETs by either inhibiting NADPH oxidase or inhibiting peptidyl arginine deiminase 4 might provide a new strategy to protect myocardial infarction injury in *ApoE^−/−^* mice. Given that most of the clinical AMI patients are accompanied by hyperglycemia or hypercholesterolemia, risk factors of NET overproduction, suppressing NET overproduction might be a target to minimize myocardial infarction injury.

## Data Availability

The data used to support the findings of this study are included within the article.

## Supplementary Materials

Supplementary Materials Figure S1: heart healing is impaired in *ApoE*-deficient mice after myocardial infarction. (a) Representative Sirius red staining and quantitation of scar size of *ApoE* ^−/−^ and WT mice 7 days after ligation. Each group involves data of 6 mice. (b) Survival curve of *ApoE* ^−/−^ (solid line) and WT mice (dotted line) after ligation (MI). Each group involves data of 15 mice (*p* < 0.05). Data are shown as mean ± SEM. Statistical tests include the two-tailed Student's *t*-test (two groups) and Log-rank test (survival curve). ^∗∗^ *p* < 0.01. Figure S2: ApoE deficiency promotes neutrophil infiltration and mobilization after myocardial infarction. (a) Representative immunofluorescent staining of Ly6G within infarcted hearts of WT and *ApoE* ^−/−^ mice 1 day after ligation. Scale bars: 100 *μ*m. (b) Serum level of TNF-*α* of *ApoE* ^−/−^ and WT mice without surgery (Ctrl) and 1 day after infarction (MI). Each group involves 8 mice. (c, d) The gating strategy of flow cytometry and the percentage of myeloid cells (CD11b^+^) and neutrophils (including monocytes) (CD11b^+^ Cr-1^+^) in the blood of *ApoE* ^−/−^ and WT mice without surgery (Ctrl) and 3 days and 7 days after ligation (MI). Each group involves 6 mice. (e) Representative immunofluorescent staining of Ly6G within infarcted hearts of *ApoE* ^−/−^ mice with or without APOE3 treatment 1 day after ligation and the percentage of the Ly6G-positive area of the whole image. Scale bars: 200 *μ*m. Each group involves 6 mice. Data are shown as mean ± SEM. Statistical tests include the two-tailed Student's *t*-test (two groups) and one-way analysis of variance followed by Tukey's multiple comparisons test (b) or by Dunnett's multiple comparison test (e). ^∗^ *p* < 0.05, ^∗∗^ *p* < 0.01, and ^&^ *p* < 0.05 (*ApoE* ^−/−^ mice vs. WT mice 3 days after ligation) and ^#^ *p* < 0.05 (*ApoE* ^−/−^ mice vs. WT mice 7 days after ligation).

## References

[1] World Health Organization (2018). *World Health Statistics 2018: Monitoring Health for the SDGs, Sustainable Development Goals*.

[2] Shen C., Ge J. (2018). Epidemic of cardiovascular disease in China. *Circulation*.

[3] Williams K. J., Tabas I. (1995). The response-to-retention hypothesis of early atherogenesis. *Arteriosclerosis, Thrombosis, and Vascular Biology*.

[4] Rasmussen K. L. (2016). Plasma levels of apolipoprotein E, APOE genotype and risk of dementia and ischemic heart disease: a review. *Atherosclerosis*.

[5] Miyata M., Smith J. D. (1996). Apolipoprotein E allele–specific antioxidant activity and effects on cytotoxicity by oxidative insults and *β*–amyloid peptides. *Nature Genetics*.

[6] Murphy A. J., Akhtari M., Tolani S. (2011). ApoE regulates hematopoietic stem cell proliferation, monocytosis, and monocyte accumulation in atherosclerotic lesions in mice. *The Journal of Clinical Investigation*.

[7] Nahrendorf M., Swirski F. K., Aikawa E. (2007). The healing myocardium sequentially mobilizes two monocyte subsets with divergent and complementary functions. *The Journal of Experimental Medicine*.

[8] Rischpler C. (2016). Acute myocardial infarction. *The Quarterly Journal of Nuclear Medicine and Molecular Imaging*.

[9] Delgado-Rizo V., Martínez-Guzmán M. A., Iñiguez-Gutierrez L., García-Orozco A., Alvarado-Navarro A., Fafutis-Morris M. (2017). Neutrophil extracellular traps and Its implications in inflammation: an overview. *Frontiers in Immunology*.

[10] Savchenko A. S., Borissoff J. I., Martinod K. (2014). VWF-mediated leukocyte recruitment with chromatin decondensation by PAD4 increases myocardial ischemia/reperfusion injury in mice. *Blood*.

[11] Ge L., Zhou X., Ji W. J. (2015). Neutrophil extracellular traps in ischemia-reperfusion injury-induced myocardial no-reflow: therapeutic potential of DNase-based reperfusion strategy. *American Journal of Physiology Heart and Circulatory Physiology*.

[12] De Meyer S. F., Suidan G. L., Fuchs T. A., Monestier M., Wagner D. D. (2012). Extracellular chromatin is an important mediator of ischemic stroke in mice. *Arteriosclerosis, Thrombosis, and Vascular Biology*.

[13] Zhang D., Chen G., Manwani D. (2015). Neutrophil ageing is regulated by the microbiome. *Nature*.

[14] Pelletier M. G., Szymczak K., Barbeau A. M., Prata G. N., O’Fallon K. S., Gaines P. (2017). Characterization of neutrophils and macrophages from ex vivo-cultured murine bone marrow for morphologic maturation and functional responses by imaging flow cytometry. *Methods*.

[15] Munoz-Caro T., Lendner M., Daugschies A., Hermosilla C., Taubert A. (2015). NADPH oxidase, MPO, NE, ERK1/2, p38 MAPK and Ca2+ influx are essential for Cryptosporidium parvum-induced NET formation. *Developmental and Comparative Immunology*.

[16] Vong L., Sherman P. M., Glogauer M. (2013). Quantification and visualization of neutrophil extracellular traps (NETs) from murine bone marrow-derived neutrophils. *Methods in Molecular Biology*.

[17] Mortaz E., Alipoor S. D., Adcock I. M., Mumby S., Koenderman L. (2018). Update on neutrophil function in severe inflammation. *Frontiers in Immunology*.

[18] Brinkmann V., Zychlinsky A. (2012). Neutrophil extracellular traps: is immunity the second function of chromatin?. *The Journal of Cell Biology*.

[19] Swirski F. K., Nahrendorf M. (2018). Cardioimmunology: the immune system in cardiac homeostasis and disease. *Nature Reviews. Immunology*.

[20] Frangogiannis N. G., Smith C. W., Entman M. L. (2002). The inflammatory response in myocardial infarction. *Cardiovascular Research*.

[21] Panizzi P., Swirski F. K., Figueiredo J. L. (2010). Impaired infarct healing in atherosclerotic mice with Ly-6C(hi) monocytosis. *Journal of the American College of Cardiology*.

[22] Ortmann W., Kolaczkowska E. (2018). Age is the work of art? Impact of neutrophil and organism age on neutrophil extracellular trap formation. *Cell and Tissue Research*.

[23] Remijsen Q., Vanden Berghe T., Wirawan E. (2011). Neutrophil extracellular trap cell death requires both autophagy and superoxide generation. *Cell Research*.

[24] Hakkim A., Fuchs T. A., Martinez N. E. (2011). Activation of the Raf-MEK-ERK pathway is required for neutrophil extracellular trap formation. *Nature Chemical Biology*.

[25] Douda D. N., Khan M. A., Grasemann H., Palaniyar N. (2015). SK3 channel and mitochondrial ROS mediate NADPH oxidase-independent NETosis induced by calcium influx. *Proceedings of the National Academy of Sciences of the United States of America*.

[26] Hou M., Wang W., Hu F., Zhang Y., Yang D., Liu Q. (2018). Phosphothreonine lyase promotes p65 degradation in a mitogen-activated protein kinase/mitogen- and stress-activated protein kinase 1-dependent manner. *Infection and Immunity*.

[27] Moccetti F., Brown E., Xie A. (2018). Myocardial infarction produces sustained proinflammatory endothelial activation in remote arteries. *Journal of the American College of Cardiology*.

[28] Rezkalla S. H., Stankowski R. V., Hanna J., Kloner R. A. (2017). Management of no-reflow phenomenon in the catheterization laboratory. *JACC: Cardiovascular Interventions*.

[29] Muthuramu I., Lox M., Jacobs F., De Geest B. (2014). Permanent ligation of the left anterior descending coronary artery in mice: a model of post-myocardial infarction remodelling and heart failure. *Journal of visualized experiments*.

[30] Wong S. L., Demers M., Martinod K. (2015). Diabetes primes neutrophils to undergo NETosis, which impairs wound healing. *Nature Medicine*.

[31] Neumann A., Brogden G., Jerjomiceva N., Brodesser S., Naim H. Y., von Köckritz-Blickwede M. (2014). Lipid alterations in human blood-derived neutrophils lead to formation of neutrophil extracellular traps. *European Journal of Cell Biology*.

