# Supplementary material for: Excessive Neutrophil Extracellular Trap Formation Aggravates Acute Myocardial Infarction Injury in Apolipoprotein E Deficiency Mice via the ROS-Dependent Pathway
Source: Oxid Med Cell Longev. 2019 May 21;2019:1209307. doi: 10.1155/2019/1209307 (PMC6556343; doi:10.1155/2019/1209307)
Supplement: Supplementary Materials — Figure S1: heart healing is impaired in ApoE-deficient mice after myocardial infarction. (a) Representative Sirius red staining and quantitation of scar size of ApoE −/− and WT mice 7 days after ligation. Each group involves data of 6 mice. (b) Survival curve of ApoE −/− (solid line) and WT mice (dotted line) after ligation (MI). Each group involves data of 15 mice (p < 0.05). Data are shown as mean ± SEM. Statistical tests include the two-tailed Student's t-test (two groups) and Log-rank test (survival curve). ∗∗ p < 0.01. Figure S2: ApoE deficiency promotes neutrophil infiltration and mobilization after myocardial infarction. (a) Representative immunofluorescent staining of Ly6G within infarcted hearts of WT and ApoE −/− mice 1 day after ligation. Scale bars: 100 μm. (b) Serum level of TNF-α of ApoE −/− and WT mice without surgery (Ctrl) and 1 day after infarction (MI). Each group involves 8 mice. (c, d) The gating strategy of flow cytometry and the percentage of myeloid cells (CD11b+) and neutrophils (including monocytes) (CD11b+ Cr-1+) in the blood of ApoE −/− and WT mice without surgery (Ctrl) and 3 days and 7 days after ligation (MI). Each group involves 6 mice. (e) Representative immunofluorescent staining of Ly6G within infarcted hearts of ApoE −/− mice with or without APOE3 treatment 1 day after ligation and the percentage of the Ly6G-positive area of the whole image. Scale bars: 200 μm. Each group involves 6 mice. Data are shown as mean ± SEM. Statistical tests include the two-tailed Student's t-test (two groups) and one-way analysis of variance followed by Tukey's multiple comparisons test (b) or by Dunnett's multiple comparison test (e). ∗ p < 0.05, ∗∗ p < 0.01, and & p < 0.05 (ApoE −/− mice vs. WT mice 3 days after ligation) and # p < 0.05 (ApoE −/− mice vs. WT mice 7 days after ligation). [file 1209307.f1.pdf]

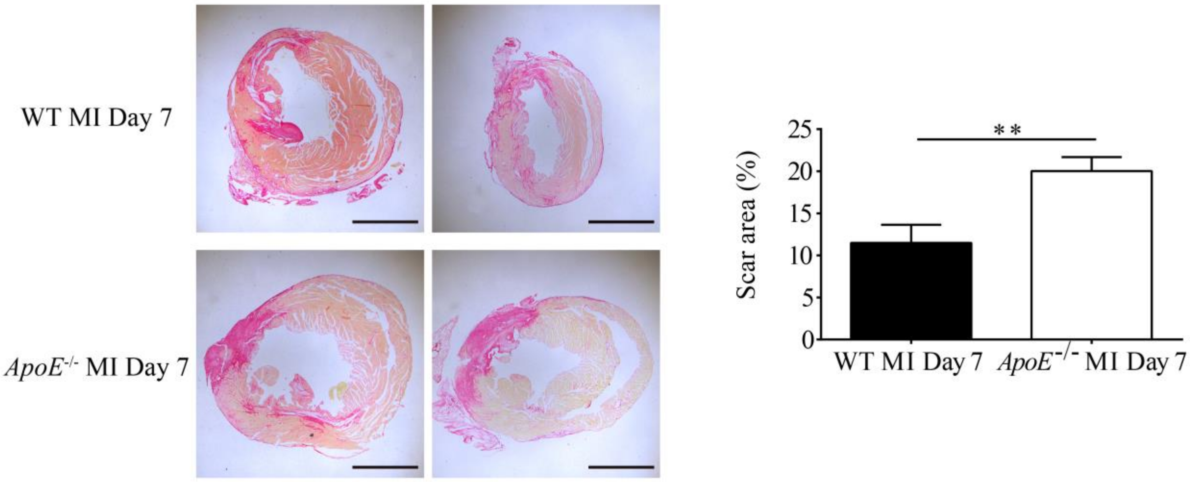

(a)

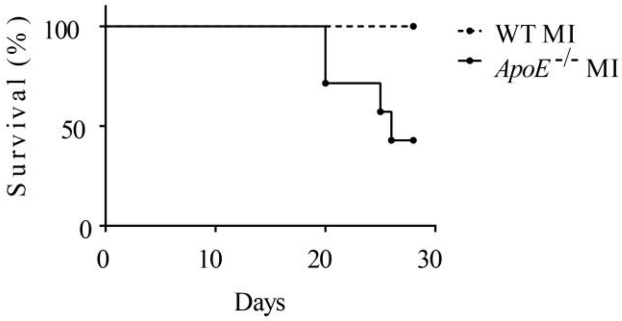

(b)

Supplementary Figure 1: Heart healing is impaired in *ApoE* deficiency mice after myocardial infarction. (a) Representative Sirius red staining and quantitation of scar size of *ApoE*$^{-/-}$ and WT mice 7 days after ligation. Each group involves data of 6 mice. (b) Survival curve of *ApoE*$^{-/-}$ (solid line) and WT mice (dotted line) after ligation (MI). Each group involves data of 15 mice, $p<0.05$. Data are shown as mean ± SEM. Statistical tests include two-tailed Student's t-test (two groups) and Log-rank test (survival curve). $^{**}p<0.01$.

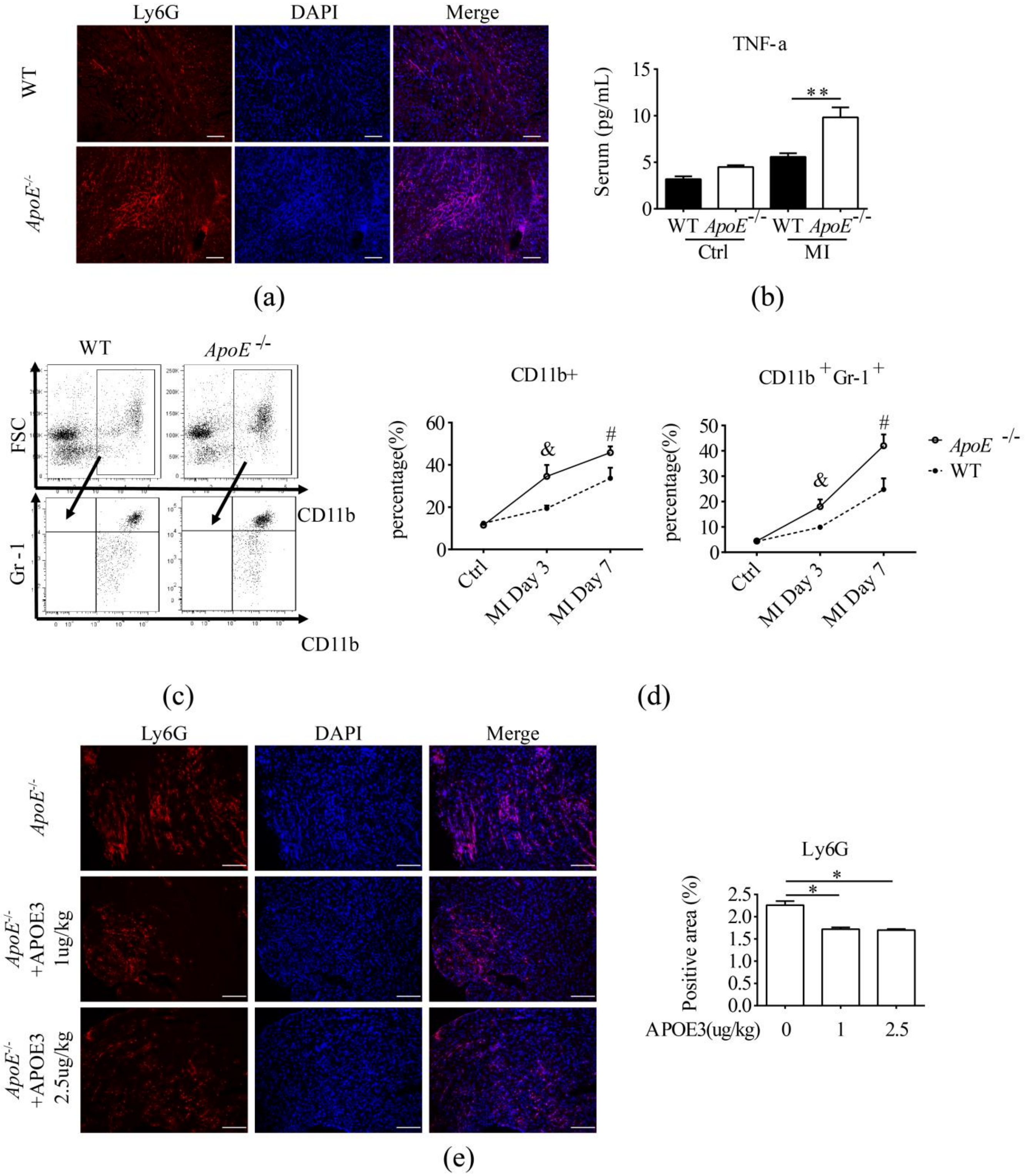

Supplementary Figure 2: ApoE deficiency promotes neutrophil infiltration and mobilization after myocardial infarction. (a) Representative immunofluorescent staining of Ly6G within infarcted hearts of WT and *ApoE*$^{-/-}$ mice 1 day after ligation. Scale bars: 100 μm. (b) Serum level of TNF-α of *ApoE*$^{-/-}$ and WT mice without surgery (Ctrl) and 1 day after

infarction (MI). Each group involves 8 mice. (c, d) The Gating strategy of flow cytometry and the percentage of myeloid cells ($CD11b^+$) and neutrophils (including monocytes) ($CD11b^+$ $Cr\text{-}1^+$) in the blood of *ApoE*$^{-/-}$ and WT mice without surgery (Ctrl) and 3 days and 7 days after ligation (MI). Each group involves 6 mice. (e) Representative immunofluorescent staining of Ly6G within infarcted hearts of *ApoE*$^{-/-}$ mice with or without APOE3 treatment 1 day after ligation and the percentage of Ly6G positive area of the whole image. Scale bars: 200 μm. Each group involves 6 mice. Data are shown as mean ± SEM. Statistical tests include two-tailed Student's t-test (two groups) and one-way analysis of variance followed by Tukey's multiple comparisons test (b) or by Dunnett's multiple comparisons test (e). *p <0.05, **p <0.01, &p<0.05 (*ApoE*$^{-/-}$ vs WT mice 3 days after ligation), #p<0.05 (*ApoE*$^{-/-}$ vs WT mice 7 days after ligation).
